# Ivy Leaf Dry Extract EA 575^®^ Is a Potent Immunomodulator Acting on Dendritic Cells

**DOI:** 10.3390/pharmaceutics17060773

**Published:** 2025-06-12

**Authors:** Miodrag Čolić, Sergej Tomić, Marina Bekić, Anđela Dubovina, Hanns Häberlein, André Rademaekers, Srđan Mašić, Dejan Bokonjić

**Affiliations:** 1 Medical Department, Serbian Academy of Sciences and Arts, 11000 Belgrade, Serbia; 2 Faculty of Medicine Foča, University of East Sarajevo, 73300 Foča, Bosnia and Herzegovina; mandicandjela21@gmail.com (A.D.); srdjan.masic@ues.rs.ba (S.M.); dejan.bokonjic@ues.rs.ba (D.B.); 3 Institute for the Application of Nuclear Energy, University of Belgrade, 11080 Belgrade, Serbia; sergej.tomic@inep.co.rs (S.T.); marina.bekic@inep.co.rs (M.B.); 4 Institute of Biochemistry and Molecular Biology, Rheinische Friedrich-Wilhelms-University of Bonn, 53115 Bonn, Germany; haeberlein@uni-bonn.de; 5 Engelhard Arzneimittel GmbH & Co. KG, 61138 Niederdorfelden, Germany; a.rademaekers@engelhard.de

**Keywords:** *Hedera helix* L., dendritic cells, cell culture, T helper cells, immunoregulation

## Abstract

**Background/Objectives**: Ivy leaf extract has been shown to alleviate bronchial infection symptoms through various mechanisms, including anti-inflammatory effects. However, its impact on adaptive immunity, particularly dendritic cell (DC)/T-cell interactions, remains unexplored. This study investigated the immunomodulatory potential of ivy leaf extract (EA 575^®^) using human monocyte-derived DCs (MoDCs). **Methods**: Immature MoDCs (imMoDCs) were differentiated with IL-4/GM-CSF and matured with LPS/IFN-γ (mMoDCs). MoDCs, treated with EA 575^®^ during differentiation, were co-cultured with purified T cells. **Results**: EA 575^®^ (non-cytotoxic up to 100 µg/mL) inhibited MoDC differentiation and maturation by reducing the expression of CD1a, CD83, CD40, CD86, HLA-DR, Dectin-1, CD206, CD209, HIF-1α, and proinflammatory cytokines (IL-12, IL-23, IL-27, IL-1β, IL-6, TNF-α). EA 575^®^-treated mMoDCs suppressed allogeneic T-cell proliferation and reduced Th1 (IFN-γ), Th17 (IL-17A, IL-22), Th9 (IL-9), Th21 (IL-21), TNF-α, and IL-6 responses. Effects were dose-dependent, with higher concentrations (100 µg/mL) showing stronger inhibition. At lower concentrations (20 µg/mL), EA 575^®^ increased Th2 (IL-4, IL-5) and IL-10 responses, and the frequencies of CD4+ T cells with Treg properties, such as CD25hiFoxp3+, Tr1 (IL-10+Foxp3−), and IL-35+ Foxp3+ cells. Immunoregulatory mechanisms mediated by EA 575^®^-treated mMoDCs correlated with the upregulation of tolerogenic markers (PD-L1, ILT3, ILT4, IDO1) on mMoDCs and the increased frequency of exhausted CD4+ T cells (PD-1+CD69+) and cytotoxic T cells (Granzyme B+PD-1+). **Conclusions**: EA 575^®^ induces tolerogenic DCs with significant anti-inflammatory and immunoregulatory properties, a previously undescribed phenomenon. Lower concentrations primarily enhance immunoregulatory responses, while higher concentrations exert more pronounced anti-inflammatory effects.

## 1. Introduction

Numerous clinical studies [1,2,3,4] have demonstrated the efficacy of ivy (*Hedera helix* L.) leaf extract in alleviating symptoms of acute and chronic respiratory tract infections by reducing coughing, bronchospasms, and secretion in both adults and children. These findings have been further substantiated by systematic reviews and meta-analyses [5,6,7]. Among the various formulations, the dry extract EA 575^®^, marketed as Prospan^®^ in liquid and tablet forms, has shown the most significant results [5,8].

Experimental studies have explored the mechanisms underlying the beneficial effects of ivy extract and its fractions. For instance, the anti-inflammatory properties of the extract were validated in a mouse model of chronic asthma [9]. Similarly, a saponin-enriched extract demonstrated efficacy in acute and chronic inflammation models in rats, likely through mechanisms involving histamine/serotonin release inhibition and macrophage modulation [10]. However, these mechanisms remain to be directly examined. Additionally, a phenolic fraction of ivy extract exhibited antimicrobial, antioxidant, and anti-inflammatory activities in a murine lipopolysaccharide (LPS)-induced acute lung injury model [11].

The anti-inflammatory effects of EA 575^®^ have also been demonstrated in vitro. In LPS-stimulated murine macrophages, EA 575^®^ significantly reduced interleukin-6 (IL-6) production in a dose-dependent manner [12]. In human monocytic and epithelial cell lines, EA 575^®^ inhibited the translocation of nuclear factor-kappa B (NF-κB) to the nucleus by stabilizing the NF-κB/inhibitor of nuclear factor-kappa B alpha (IκBα) complex and reducing IκBα phosphorylation [13]. Since NF-kB regulates genes encoding pro-inflammatory cytokines [14], including interleukin (IL)-6, this pathway represents a key target of EA 575^®^. Furthermore, EA 575^®^ was shown to block adenosine receptor A2B signaling, enhancing β2-adrenergic responsiveness and reducing IL-6 production, which likely contributes to its bronchodilatory and anti-inflammatory effects [15].

The bioactive components responsible for the health benefits of ivy leaf extract are predominantly triterpene saponins, such as hederacosides A, B, and C, and α-hederin, along with polyphenols, flavonoids, alkaloids, and other constituents [16,17]. Both saponins [18] and polyphenols [19] possess anti-inflammatory properties through mechanisms like the direct inhibition of pro-inflammatory cytokine production and modulation of arachidonic acid metabolism. Polyphenols also stimulate natural killer (NK) cells and promote anti-inflammatory cytokines [20].

Despite these findings, the effect of *Hedera helix* L. extract on T cell-mediated immunity remains largely unexplored. Studies of its constituents, isolated from other plants or used in purified forms, suggest potential T-cell modulatory effects [21,22]. Notably, saponins exhibit strong adjuvant activity, enhancing T cell-mediated responses, including CD8+ cytotoxicity [23]. Polyphenols are also potent immunomodulators, but their effects depend on various factors, such as the source and extraction method [24].

This study aims to investigate the immunomodulatory potential of EA 575^®^ using an in vitro model of monocyte-derived dendritic cells (MoDCs) co-cultured with T cells. As potent antigen-presenting cells, DCs initiate and regulate T cell-mediated immunity [25]. MoDCs, specifically “inflammatory MoDCs”, play a critical role in inflammation [26] and infection [27]. The DC/T-cell co-culture model enables the evaluation of DC differentiation, maturation, alloreactivity, Th polarization, and T regulatory cell (Treg) induction [28].

## 2. Materials and Methods

### 2.1. Characterization of EA 575^®^ by HPLC Analysis

Ivy leaf dry extract EA 575^®^ (DER 5-7.5:1, 30% ethanol) (Batch number 21N0160) was obtained from Engelhard Arzneimittel GmbH & Co. KG (Niederdorfelden, Germany). EA 575^®^ (10 mg) was dissolved in 5 mL of 50% EtOH, passed through a 0.45 µm filter, and analyzed using a high-performance liquid chromatography (HPLC) system (Agilent Series 1200 HPLC system, Agilent Technologies, Inc., Santa Clara, CA, USA) equipped with a YMC-Triart C18 column, 250 × 4.6 mm I.D., S-5 µm, 12 nm (YMC Separation Technology, YMC Co., Ltd., Kyoto, Japan), and a photodiode array detector (200–500 nm). The mobile phases were as follows: Solvent A: H_2_O/acetonitrile (44:2, *v*/*v*), pH adjusted to 2.0 with 85% phosphoric acid; Solvent B: acetonitrile. The following linear gradient was used: 0–9 min 100% A, 9–10 min to 94% A, 10–25 min to 85% A, 25–50 min to 40% A, 50–51 min to 0% A, 51–65 min 0% A. The flow rate was 1 mL/min up to 50 min. From 51 to 65 min, the flow rate was 1.5 mL/min. The analysis was performed over a detection period of 0–65 min at 205 nm.

Identification of constituents was performed by comparing the UV spectra of reference substances and their corresponding retention times. Chromatograms were recorded and evaluated using Agilent ChemStation Software Version B.04.01. The following ingredients were identified: protocatechuic acid, neochlorogenic acid, chlorogenic acid, cryptochlorogenic acid, rutin, kaempferol-3-O-rutinoside, 3,4-, 3,5-, and 4,5 dicaffeoylquinic acid, hederacoside B, C, D, and F, hederaginin-3-O-glucoside, α-, β-, and δ-hederin. All data about their identification and estimated and calculated contents are presented in previous papers [11,29]. This is under the requirements of the *European Pharmacopoeia*: the quality of ivy leaf is determined by hederacoside C (*European Pharmacopoeia* 10, 2019).

The following compounds, that are used as reference standards, were sourced from Phytolab (Vestenbergsgreuth, Germany): 3,4-, 3,5-, and 4,5-dicaffeoylquinic acid, protocatechuic acid, chlorogenic acid, neochlorogenic acid, and cryptochlorogenic acid. Rutin, kaempferol-3-O-rutinoside, and hederins were purchased from HWI (Rülzheim, Germany), while hederacosides were supplied by ChromaDex (Irvine, CA, USA).

### 2.2. Cell Cultures

All experiments on human cells were approved by the Ethical Board of the University of East Sarajevo, Medical Faculty Foca, and were conducted according to institutional guidelines. Peripheral blood mononuclear cells (PBMCs) were isolated from citrated venous blood taken from healthy volunteers using density gradient centrifugation on lymphocyte separation medium 1077 (PAA, Linz, Austria). Blood donors provided written informed consent by the Declaration of Helsinki. The cells from the interphase were collected, washed three times by low-speed centrifugation to remove platelets, and subjected to immunomagnetic-activated cell sorting (MACS) (Miltenyi Biotec, Bergisch Gladbach, Germany) to purify monocytes (CD14+ cells) and T (CD3+) cells. Monocytes were isolated using the Monocyte Isolation Kit II, and T cells were isolated using the Pan T cell Isolation Kit, following the manufacturer’s instructions (Miltenyi Biotec, Bergisch Gladbach, Germany). The purity of the isolated cells was approximately 92% for monocytes and 95% for T cells.

Monocytes (0.5 × 10⁶/mL) were cultured in 6-well plates (Sarstedt, Nümbrecht, Germany) for 4 days under standard culture conditions. Dendritic cell (DC)-specific growth medium, obtained from CellGenix, Freiburg, Germany, was supplemented with human recombinant Granulocyte Macrophage-Colony Stimulating Factor (GM-CSF) (Novartis, Basel, Switzerland) (100 ng/mL) and human recombinant IL-4 (Roche Diagnostics, Basel, Switzerland) (25 ng/mL). This 4-day protocol reliably yielded immature monocyte-derived DCs (imMoDCs). The treatment of imMoDCs with 50 ng/mL interferon-γ (IFN-γ) (R&D Systems, Minneapolis, MN, USA) and 200 ng/mL lipopolysaccharide (LPS) from *Escherichia coli* 0.111:B4 (Sigma-Aldrich, Darmstadt, Germany) for 24 h resulted in the generation of mature MoDCs (mMoDCs). EA 575^®^ (20 µg/mL and 100 µg/mL) was added either to monocyte cultures at the beginning of imMoDC differentiation or before maturation induction. On day 5, both control and EA 575^®^-treated imMoDCs and mMoDCs were harvested, washed twice with RPMI-1640 medium (Sigma-Aldrich, Darmstadt, Germany), counted, and used for downstream applications. Culture supernatants were collected and stored at −80 °C for later cytokine analysis.

### 2.3. Cytotoxicity Assays

The cytotoxic potential of EA 575^®^ was evaluated using an MTT assay and an apoptosis/necrosis assay. Monocytes (1.5 × 10^5^ cells/well), undergoing differentiation into imMoDCs, were seeded in 96-well flat-bottom plates (Sarstedt, Nümbrecht, Germany) and cultured in a complete DC culture medium for 24 or 48 h. Triplicates of each culture were treated with doubling concentrations of EA 575^®^ (12.5–400 µg/mL), whereas untreated cultures served as controls. Cell viability was assessed by incubating the cultures with MTT reagent (3-(4,5-dimethylthiazol-2-yl)-2,5-diphenyltetrazolium bromide) at a final concentration of 0.5 mg/mL for 4 h. Parallel cell-free wells containing corresponding concentrations of EA 575^®^ served as blank controls. After incubation, formazan crystals were solubilized by overnight treatment with a solution containing 10% (*w*/*v*) sodium dodecyl sulfate (SDS; Millipore, Burlington, MA, USA) and 0.01 N (*v*/*v*) hydrochloric acid (HCl; Sigma-Aldrich, Darmstadt, Germany). Absorbance was measured at 570 nm and 670 nm using an ELx800 microplate reader (Biotek, Winooski, VT, USA). Corrected optical density (OD) values were calculated by subtracting the blank values to obtain net readings. The percentage of relative metabolic activity (MTT%) in treated cultures was determined by normalizing to untreated control cells, which were set as 100%.

To investigate the mode of cell death, including apoptosis and necrosis, monocytes differentiating into imMoDCs were cultured under the same conditions for 24 or 48 h, followed by staining with Annexin V-FITC and Propidium Iodide (PI) using a commercially available kit (R&D Systems, Minneapolis, MN, USA), according to the manufacturer’s protocol. Briefly, 2 × 10^5^ cells per tube were resuspended in calcium-containing binding buffer and then incubated with Annexin V-FITC and PI. The fluorescence was analyzed using a BD LSR II flow cytometer (BD Biosciences, Franklin Lakes, NJ, USA). Data were processed offline using FCS Express 7.26 (De Novo Software, CA, USA). Based on staining patterns, cells were classified as early apoptotic, late apoptotic/secondary necrotic, or primarily necrotic.

Additionally, overall cell viability at the end of the 5-day differentiation period was assessed in both imMoDC and mMoDC cultures by staining the cells with 1% Trypan Blue solution and observing them under a light microscope. Viability was determined using the formula:

Viability (%) = (Number of viable cells on day 5/Number of initially seeded cells) × 100.

### 2.4. Autophagy and Reactive Oxygen Species Assays

Autophagy was analyzed by measuring autophagy flux. Monocytes, induced to differentiate into imMoDCs, were cultured with 20 or 100 µg/mL of EA 575^®^ for 24 h as described above. At the end of the culture period, the cells were collected, washed twice with PBS, and stained using a Muse Autophagy Kit (Luminex Corporation, Austin, TX, USA). The kit detects a membrane-converted variant of LC3, known as LC3-II. The cells were stained either directly with an anti-LC3-II antibody or, alternatively, washed first with phosphate-buffered saline (PBS) and incubated for 4 h in PBS with bafilomycin, according to the manufacturer’s protocol. The total expression of LC3-II was determined using a Guava Muse Cell Analyzer (Luminex). Autophagy flux was calculated as the ratio of bafilomycin-treated cells to non-treated cells in each culture.

To evaluate the production of reactive oxygen species (ROS), a Muse Oxidative Stress Kit (Luminex Corporation) was used. In brief, monocytes induced to differentiate into imMoDCs were cultured with 20 or 100 µg/mL of EA 575^®^ for 24 h, as described above. After 24 h, the cells were collected, washed with PBS, and incubated with dihydroethidium (DHE) dye, which serves as a probe for ROS detection. The stained cells were analyzed using a Guava Muse Cell Analyzer (Luminex).

### 2.5. Alloreactivity Assay

The ability of EA 575^®^-treated MoDCs to stimulate T-cell proliferation was evaluated using an allogeneic Mixed Leukocyte Reaction (MLR). Purified allogeneic CD3^+^ T cells (1 × 10^5^ cells/well) were labeled with carboxyfluorescein diacetate succinimidyl ester (CFSE; Invitrogen, Waltham, MA, USA) and co-cultured with imMoDCs or mMoDCs at various dendritic cell-to-T-cell ratios (1:10 to 1:40) in a complete RPMI 1640 medium consisting of RPMI 1640, 10% fetal calf serum (FCS), 50 µM 2-mercaptoethanol, and antibiotics (penicillin and streptomycin, both at 100 U/mL). All reagents were obtained from Sigma-Aldrich, Darmstadt, Germany.

After 4 days of co-culture, cells were harvested and stained with 7-aminoactinomycin D (7-AAD; 50 µg/mL, Sigma-Aldrich, Darmstadt, Germany) to exclude dead cells from analysis. T-cell proliferation was assessed by flow cytometry (BD Biosciences, Franklin Lakes, NJ, USA) based on CFSE dilution, with doublets excluded during gating. Data were analyzed offline using FCS Express 7.26 (De Novo Software, CA, USA).

To evaluate cytokine production, co-cultures were restimulated with 20 ng/mL phorbol 12-myristate 13-acetate (PMA) and 500 ng/mL ionomycin (both from Sigma-Aldrich, Darmstadt, Germany) for 6 h, followed by supernatant collection for subsequent cytokine analysis. In parallel cultures, intracellular cytokine expression was assessed by adding monensin (2 mM, Sigma-Aldrich) 4 h before cell harvesting.

### 2.6. T Helper Polarization Assay

To study Th polarization capabilities, control and EA 575^®^-treated mMoDCs were co-cultured with purified T cells at a 1:20 MoDC/T-cell ratio for 5 days in round-bottom 96-well plates. The culture medium used was the complete RPMI medium. After the culture period, the cells were treated with PMA and ionomycin as described above to collect either cell-free supernatants or cells. The collected cells were then processed for flow cytometry, and the supernatants were frozen at −80 °C until cytokine measurement.

### 2.7. Induction of T Regulatory and Exhausted Cells

To induce regulatory T cells (Tregs), and exhausted T cells, co-cultures of mature MoDCs (mMoDCs) and allogeneic CD3^+^ T cells were established at a suboptimal mMoDC-to-T-cell ratio of 1:50. Cultures were maintained for 6 days in complete RPMI 1640 medium supplemented with 2 ng/mL recombinant human interleukin-2 (IL-2; R&D Systems, Minneapolis, MN, USA). On day 6, the co-cultures were stimulated with PMA, ionomycin, and monensin as described in Section 2.5, followed by flow cytometric analysis to evaluate Tregs and exhausted T cells based on characteristic marker expression.

### 2.8. Cytokine Assays

Cytokine profiling in cell culture supernatants was performed using both multiplex and ELISA-based methods. A panel of cytokines, including IL-2, IL-6, IL-9, IL-10, IL-12p70, IL-12p17, IL-13, IL-17A, IL-21, IL-22, IL-23, IL-27, TNF-α, and IFN-γ, was simultaneously quantified using a LEGENDplex™ Flow Cytomix bead-based assay (BioLegend, San Diego, CA, USA), following the protocol provided with the Cytomix kit. Additionally, selected cytokines such as IL-1β, IL-12p70, IL-23, IL-27, and transforming growth factor-beta (TGF-β) were quantified using specific sandwich ELISA kits (R&D Systems, Minneapolis, MN, USA). For all cytokine measurements, concentrations were calculated based on standard curves generated using known cytokine standards. Results were normalized to the number of cells: 3 × 10^5^ MoDCs for MoDC-only cultures and 1 × 10^5^ T cells for MoDC/T-cell co-cultures.

### 2.9. Flow Cytometry

To study the phenotype, MoDCs and T cells were stained with fluorochrome-conjugated monoclonal antibodies (mAbs) targeting surface and intracellular markers, alongside appropriate isotype controls. Fluorescence-minus-one (FMO) controls were used to ensure specificity and gating accuracy.

Monoclonal antibodies were obtained from the following suppliers:

- Bio-Rad (Hercules, CA, USA): IgG1-PE (MCA928PE), IgG1-FITC (MCA928F).
- BioLegend (Basel, Switzerland): anti-CD1a-PerCP/Cy5.5 (HI149), HLA-DR-APC/Cy7 (L243), IL-4-PerCP/Cy5.5 and -PE (MP4-25D2), ILT-4-APC (42D1), CD25-PE (BC96), CD25-PerCP/Cy5.5 (M-A251), IL-10-APC and -PE (JES5-16E3), TGF-β-APC (TW4-6H10), IL-17A-Alexa Fluor 488 (BL168), IFN-γ-APC and -FITC (4S.B3), CD83-FITC (HB15e), PD-L1-PE (29E.2A3), IgG1-PerCP/Cy5.5 (HTK888).
- R&D Systems (Minneapolis, MN, USA): CD3-FITC and -PE, CD4-FITC and -APC (11830), IL-12p40/p70-PE (C11.5), TGF-β-PE (9016), IDO-1-APC (700838).
- Miltenyi Biotec (Gladbach, Germany): CD14-FITC (TUK4), IL-1β-PE (REA1172).
- Thermo Fisher Scientific (Dreieich, Germany): CD86-PE (IT2.2), streptavidin-PerCP and -APC, ILT3-PE (ZM4.1), IgG1-APC (MA5-18093), IL-17A-APC (eBio17B7).
- BD Biosciences (San Diego, CA, USA): CD40-APC (5C3), CD69-PE, FoxP3-PerCP/Cy5.5 and -Alexa Fluor 488 (236A/E7), granzyme B-APC, CD33-APC, IL-35-PerCP, PD-1-FITC.
- Elabscience (Houston, TX, USA): CD8-PerCP/Cy5.5 (HIT8a).

For intracellular staining, surface-labeled cells were fixed and permeabilized using a BioLegend Fixation and Permeabilization Kit. Samples were acquired on a BD LSR II flow cytometer (BD Biosciences, Franklin Lakes, NJ, USA). Compensation for spectral overlap was performed using single-stained controls. Dead cells and doublets were excluded based on FSC-A/SSC-A and FSC-H parameters, respectively. A minimum of 10,000 events per sample was analyzed. Data were processed using FCS Express 7.26 (De Novo Software, CA, USA) and expressed either as the percentage of positive cells or mean fluorescence intensity (MFI).

### 2.10. Statistical Analysis

The results are presented as representative data or as the mean ± SD from six independent experiments conducted with six donors. Initially, the normality of the data was tested using a Shapiro–Wilk test. Differences in the mean values between the control and experimental cultures were analyzed using a one-way ANOVA with a Dunnett’s post-test. Statistical analyses were performed using GraphPad Prism software version 8.1 (GraphPad, La Jolla, CA, USA). Values of *p* < 0.05 were considered statistically significant.

## 3. Results

### 3.1. Cytotoxicity of EA 575^®^

**Figure 1:**
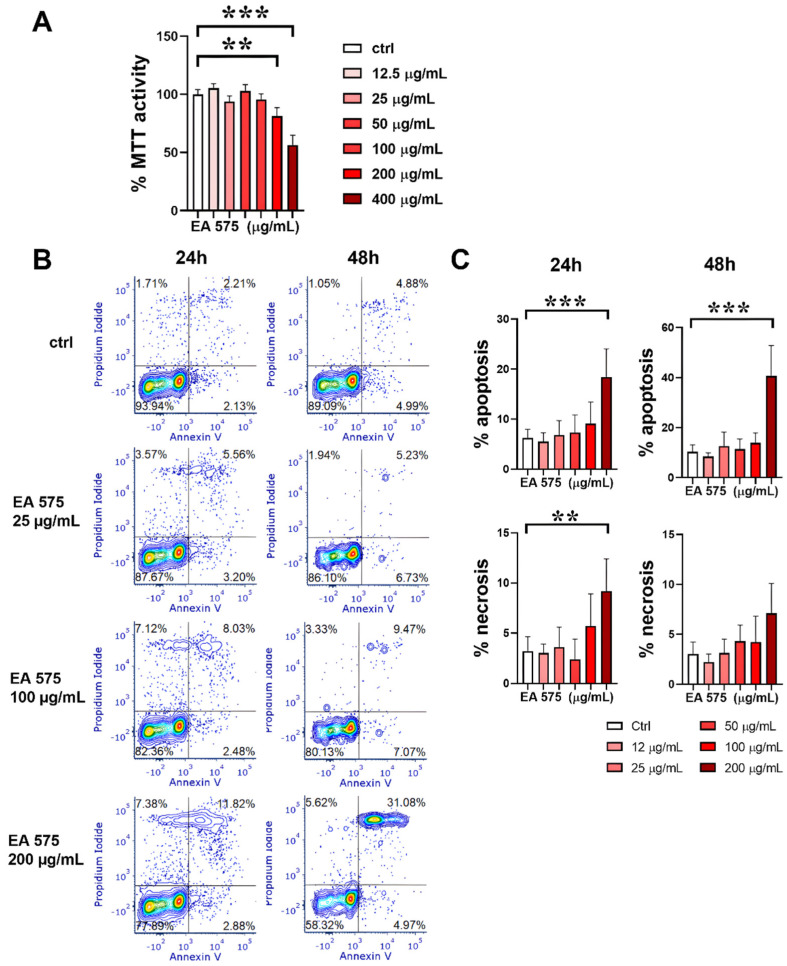
The effect of EA 575^®^ on the cytotoxicity of monocytes induced to differentiate into imMoDCs. (**A**) Metabolic activity assessed by the MTT assay. Values are expressed as the mean ± SD (*n* = 4). (**B**) Representative flow cytometry plots from one experiment showing apoptosis and necrosis. (**C**) Apoptosis and necrosis presented as the mean ± SD (*n* = 4). ** *p* < 0.01; *** *p* < 0.001 compared to the control (Ctrl).

The first set of experiments aimed to evaluate the cytotoxicity of EA 575^®^ using monocytes at the initial stage of DC differentiation. The initial screening was performed using the MTT assay. The results presented in Figure 1A indicate that EA 575^®^, at concentrations up to 100 µg/mL, did not significantly affect cellular metabolic activity. However, concentrations of 200 µg/mL and 400 µg/mL reduced metabolic activity (*p* < 0.01 and *p* < 0.001, respectively), indicating a cytotoxic effect at higher concentrations.

To investigate the mode of cell death, an apoptosis/necrosis assay was conducted. Figure 1B,C demonstrate that 200 µg/mL of EA 575^®^ induced apoptosis in both cell types, with a further increase observed after 48 h (*p* < 0.001). Additionally, the highest concentration slightly increased the proportion of necrotic cells after 24 h (*p* < 0.01).

The highest non-cytotoxic concentration of the extract (100 µg/mL) triggered ROS production without significantly affecting autophagy (Supplementary Figure S1).

### 3.2. EA 575^®^ Impairs the Differentiation of MoDCs

**Figure 2:**
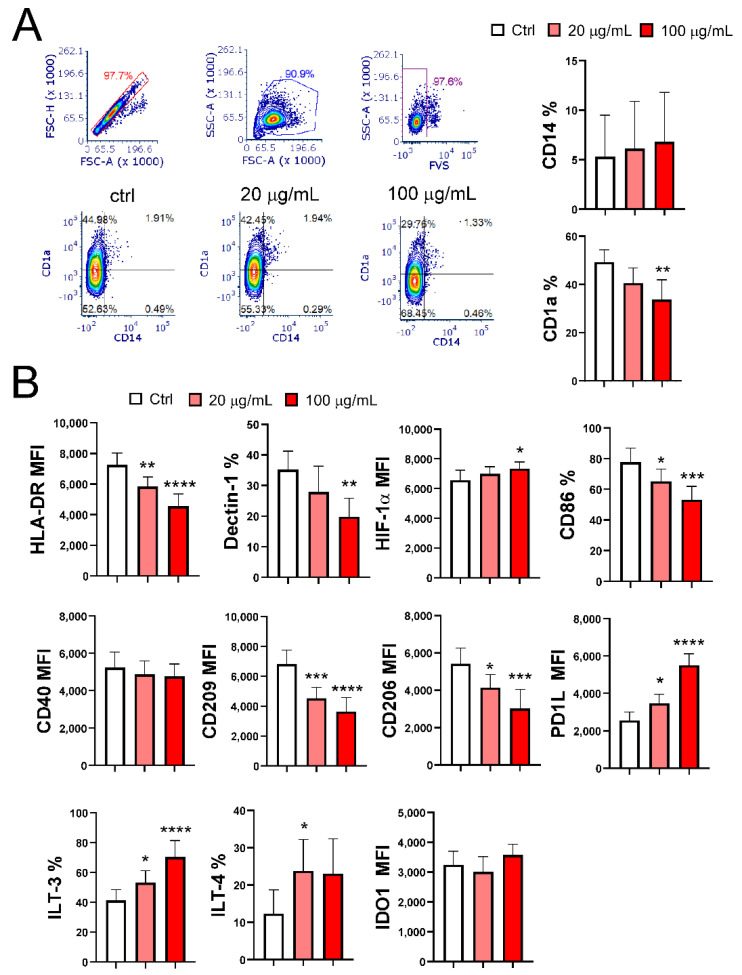
The effect of EA 575^®^ on the expression of differentiation markers on imMoDCs. MoDCs were differentiated from monocytes in the presence of lower (20 µg/mL) and higher (100 µg/mL) concentrations of EA 575^®^ over 5 days. (**A**) Expression of CD1a and CD14: representative flow cytometry plots from one experiment; histograms show the mean percentages of positive cells ± SD (*n* = 6 donors). (**B**) Expression of differentiation markers presented as mean percentages or mean fluorescence intensity (MFI) ± SD (*n* = 6 donors). * *p* < 0.05; ** *p* < 0.01; *** *p* < 0.001; **** *p* < 0.0001 compared to the control (Ctrl).

To study the effect of EA 575^®^ on the differentiation of MoDCs, monocytes were treated with low (20 µg/mL) and high (100 µg/mL) non-cytotoxic concentrations of EA 575^®^ at the beginning of MoDC differentiation. Phenotypic analysis, performed on day 5, showed a significant impairment in imMoDC differentiation, with the effect being more pronounced at the higher concentration (Figure 2A,B). This conclusion is based on the lower expression of CD1a (*p* < 0.01), Dectin-1 (*p* < 0.01), CD206 (*p* < 0.001), CD209 (*p* < 0.0001), CD86 (*p* < 0.001), and HLA-DR (*p* < 0.0001) compared to control imMoDCs. In contrast, the expression of CD40 remained unchanged, while HIF-1α expression was upregulated (*p* < 0.05). Additionally, CD14 was nearly completely downregulated in both control and EA 575^®^-treated MoDCs. The expression of these markers on imMoDCs in the presence of a lower concentration of EA 575 was either not significantly different compared to the control (CD1a, Dectin-1, and HIF-1α) or less pronounced compared to the higher concentration of EA 575 for the following markers: CD206 (*p* < 0.05), CD209 (*p* < 0.001), CD86 (*p* < 0.05), and HLA-DR (*p* < 0.01).

Regarding tolerogenic markers, the expression of programmed death-ligand 1 (PD-L1) and immunoglobulin-like transcript 3 (ILT3) increased in a dose-dependent manner (*p* < 0.05 and *p* < 0.0001, respectively), while ILT4 expression was upregulated only in the presence of the lower concentration of EA 575^®^ (*p* < 0.05). In contrast, Indoleamine 2,3-Dioxygenase 1 (IDO1) expression remained unchanged.

ImMoDCs differentiated in the presence of EA 575^®^, similar to control imMoDCs, produced low levels of most cytokines, except IL-6, IL-8, and IL-27. IL-27 was significantly upregulated by EA 575^®^ at higher concentrations (*p* < 0.001). Conversely, the same concentration of the extract reduced the production of IL-6 (*p* < 0.001), IL-8 (*p* < 0.0001), and tumor necrosis factor (TNF)-α (*p* < 0.0001). No significant changes were observed in the levels of transforming growth factor (TGF-β), IL-12, IL-23, and IL-10 (Supplementary Table S1).

### 3.3. EA 575^®^ Impairs the Phenotypic Maturation of MoDCs

Control and EA 575^®^-treated imMoDCs, differentiated for 4 days, were induced to mature using LPS and IFN-γ, as described in the Section 2. On day 5, MoDCs were analyzed for the expression of several membrane-bound and intracellular markers. As shown in Supplementary Table S2, LPS and IFN-γ stimulation promoted the complete maturation of control MoDCs, evidenced by the upregulated expression of CD83 (a maturation marker), CD86 and CD40 (costimulatory molecules), and HLA-DR. Conversely, the expression of markers associated with antigen recognition (CD206 and CD209) was downregulated compared to control imMoDCs.

**Figure 3:**
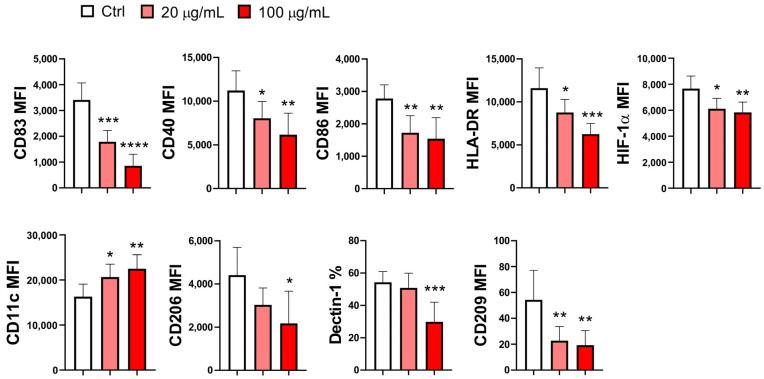
The effect of EA 575^®^ on the expression of differentiation/maturation markers on mMoDCs. MoDCs were differentiated from monocytes in the presence of lower (20 µg/mL) and higher (100 µg/mL) concentrations of EA 575^®^ for 4 days and then induced to mature with LPS/ IFN-γ. The expression of markers on mMoDCs was presented as mean percentages or mean fluorescence intensity (MFI) ± SD (*n* = 6 donors). * *p* < 0.05; ** *p* < 0.01; *** *p* < 0.001, ****p < 0.0001 compared to the control (Ctrl).

The maturation of MoDCs differentiated in the presence of EA 575^®^ was significantly inhibited compared to control mMoDCs, as indicated by a decrease in the expression of nearly all markers. This effect was dose-dependent for CD83 (*p* < 0.001 and *p* < 0.0001), CD40 (*p* < 0.05 and *p* < 0.01), HLA-DR (*p* < 0.05 and *p* < 0.001), and HIF-1α (*p* < 0.05 and *p* < 0.01). No dose-dependent differences were observed for CD86 (*p* < 0.01) and CD209 (*p* < 0.01), where the effects were similar at both concentrations. The expression of CD206 and Dectin-1 was inhibited only at the higher concentration of EA 575^®^ (*p* < 0.05 and *p* < 0.001, respectively). In contrast, the expression of CD11c, a conventional DC marker, was downregulated in a dose-dependent manner (*p* < 0.05 and *p* < 0.01, respectively) (Figure 3 and Supplementary Figure S2).

**Figure 4:**
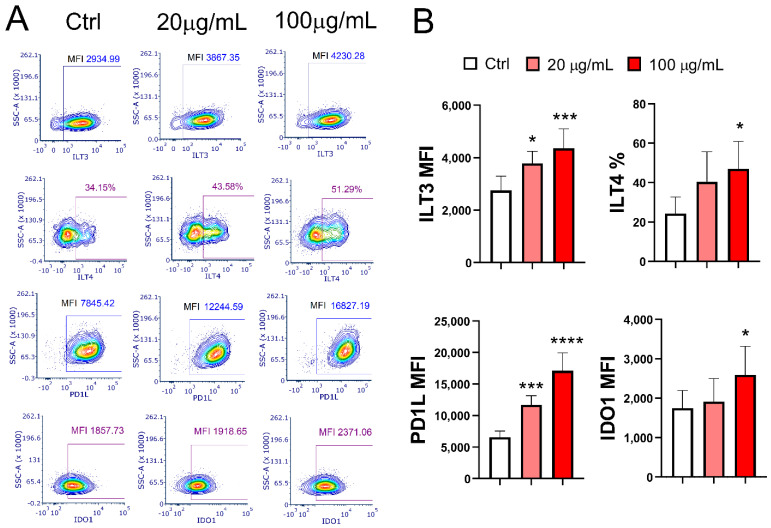
The effect of EA 575^®^ on the expression of tolerogenic markers on mMoDCs. MoDCs were differentiated from monocytes in the presence of lower (20 µg/mL) and higher (100 µg/mL) concentrations of EA 575^®^ for 4 days and then induced to mature with LPS/ IFN-γ. The expression of markers on mMoDCs was examined on day 5 and presented as (**A**) percentages or mean fluorescence intensity (MFI) of one representative experiment or (**B**) mean percentages or MFI ± SD (*n* = 6 donors). * *p* < 0.05; *** *p* < 0.001, ****p < 0.0001 compared to the control (Ctrl).

The expression of tolerogenic markers PD-L1 (*p* < 0.001 and *p* < 0.0001) and ILT3 (*p* < 0.05 and *p* < 0.001) on mMoDCs was upregulated in a dose-dependent manner in the presence of EA 575^®^. In contrast, the expression of ILT4 (*p* < 0.05) and IDO1 (*p* < 0.05) was upregulated only at the higher concentration of EA 575^®^ (Figure 4).

### 3.4. Effect of EA 575^®^ on the Production of Cytokines by MoDCs

The production of cytokines relevant for immune response stimulation in the supernatants of mMoDC cultures, including IL-12p70 (*p* < 0.0001), IL-23 (*p* < 0.0001), IL-27 (*p* = 0.0001), IL-1β (*p* < 0.01 and *p* < 0.001, respectively), and TNF-α (*p* < 0.05 and *p* < 0.0001, respectively), was significantly downregulated by EA 575^®^ in a dose-dependent manner.

**Figure 5:**
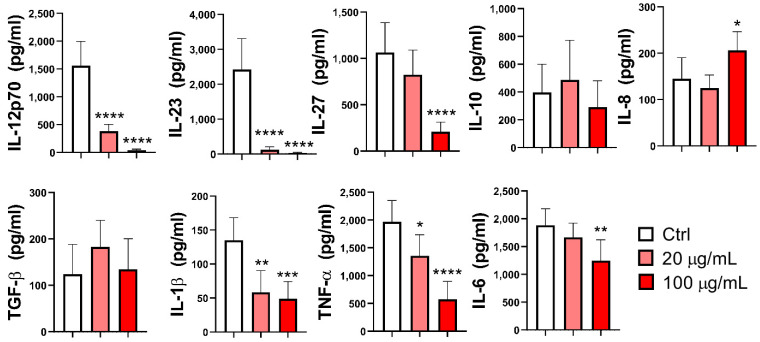
The effect of EA 575^®^ on the production of cytokines by mMoDCs. MoDCs were differentiated from monocytes in the presence of lower (20 µg/mL) and higher (100 µg/mL) concentrations of EA 575^®^ for 4 days and then induced to mature with LPS/ IFN-γ. Levels of cytokines were presented as the mean pg/mL standardized to 3 × 10^5^ MoDCs ± SD (*n* = 6 donors). * *p* < 0.05; ** *p* < 0.01; *** *p* < 0.001; **** *p* < 0.0001 compared to the control (Ctrl).

The production of IL-27 (*p* < 0.0001) and IL-6 (*p* < 0.01) was reduced only in the presence of a higher concentration of EA 575^®^. The production of immunoregulatory cytokines IL-10 and TGF-β remained unchanged, whereas the level of IL-8 was significantly increased (*p* < 0.05) at the higher concentration of EA 575^®^ (Figure 5). Supplementary Table S2 provides a summary overview of the effects of EA 575^®^ on cytokine production by imMoDCs and mMoDCs.

A similar dose-dependent effect was observed at the intracellular level for cytokines such as IL-12p70 (*p* < 0.001 and *p* < 0.0001, respectively), IL-1β (*p* < 0.05 and *p* < 0.01, respectively), and TNF-α (*p* < 0.05 and *p* < 0.01, respectively). In contrast, the frequency of IL-10+ cells was significantly increased by both EA 575^®^ concentrations (*p* < 0.001), while the expression of IL-33 and TGF-β remained unchanged (Supplementary Figure S3).

### 3.5. Effect of EA 575^®^-Treated MoDCs on the Proliferation of Alloreactive T Cells

**Figure 6:**
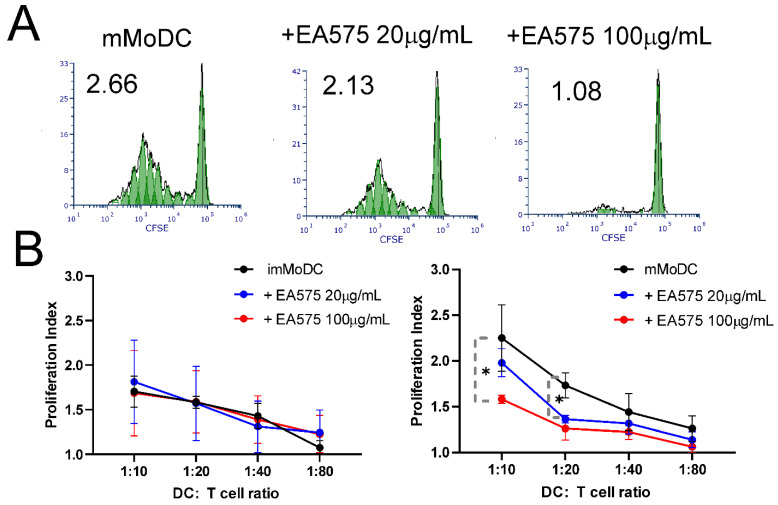
The effect of EA 575^®^-treated MoDCs on the proliferation of allogeneic T cells. Control and EA 575^®^-treated im- or mMoDCs were incubated with CFSE-prelabeled T cells at different ratios. Proliferation was measured by flow cytometry as described in the Section 2. The upper row (**A**) presents histograms of proliferating cells and corresponding indexes of one representative experiment. (**B**) Proliferation indexes (mean ± SD; *n* = 3). * *p* < 0.05 compared to the corresponding control.

MoDCs were co-cultured with purified allogeneic T cells previously stained with the CFSE fluorescent dye at varying MoDC/T-cell ratios. After 4 days, proliferation indices were determined based on the dilution of the fluorescent dye resulting from the doubling of proliferating cell populations. The results shown in Figure 6 indicate that mMoDCs pretreated with EA 575^®^ during differentiation induced a dose-dependent inhibition of T-cell proliferation. However, the effect was statistically significant only at MoDC/T-cell ratios of 1:10 and 1:20. As expected, imMoDCs had no effect, regardless of the concentration of the extract.

### 3.6. Effect of EA 575^®^-Treated MoDCs on Th Polarization

**Figure 7:**
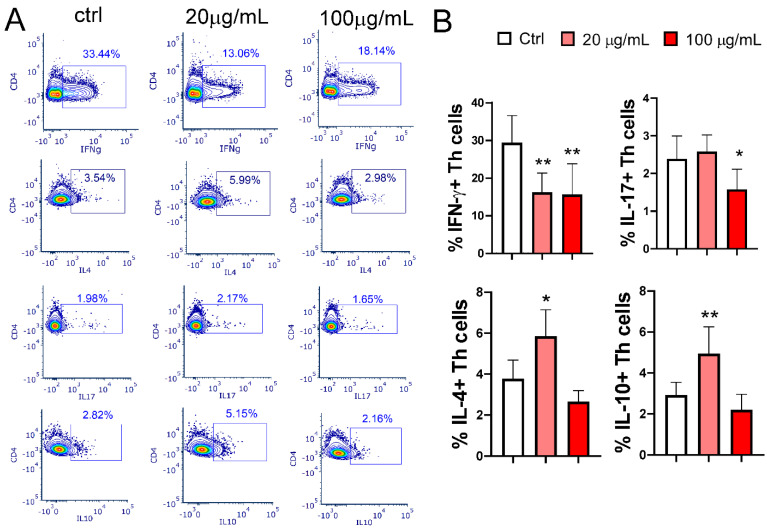
The effect of EA 575^®^-treated MoDCs on Th polarization. Control and EA 575^®^-treated mMoDCs were co-cultured with purified T cells as described in the Section 2. Th polarization was assessed by flow cytometry based on the expression of intracellular cytokines. (**A**) Representative plots from one experiment. (**B**) Mean percentages of positive cells ± SD (*n* = 6 donors). * *p* < 0.05; ** *p* < 0.01 compared to the control (Ctrl).

EA 575^®^-treated MoDCs significantly modulate Th polarization. The effect of the extract on mMoDCs pretreated with the lower concentration of EA 575^®^ during differentiation was more pronounced, as evidenced by a decreased frequency of IFN-γ+CD4+ (Th1) cells (*p* < 0.01) alongside an increased frequency of IL-4+CD4+ (Th2) cells (*p* < 0.05) and IL-10+CD4+ T cells (*p* < 0.01). MoDCs pretreated with a higher concentration of EA 575^®^ reduced the frequency of Th1 (*p* < 0.01) and Th17 cells (*p* < 0.05) without significantly affecting Th2 or IL-10+CD4+ T cells (Figure 7).

**Figure 8:**
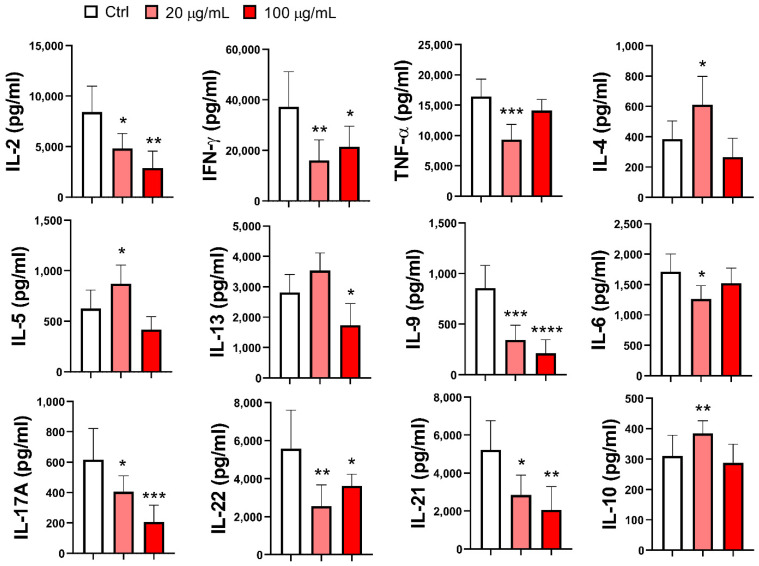
The effect of EA 575^®^-treated mMoDCs on Th polarization, determined by cytokine levels in co-culture supernatants. Control and EA 575^®^-treated mMoDCs were co-cultured with purified T cells as described in the Section 2. Th polarization was assessed by measuring cytokine production in the supernatants of mMoDC/T-cell co-cultures using flow cytometry. Values are expressed as mean cytokine concentrations (pg/mL) ± SD (*n* = 6 donors). * *p* < 0.05; ** *p* < 0.01; *** *p* < 0.001; **** *p* < 0.0001 compared to the control (Ctrl).

A much larger panel of cytokines was analyzed in the supernatants of mMoDC/T-cell co-cultures. As shown in Figure 8, the production of Th1 cytokines (IFN-γ) and Th1-related cytokines (IL-2 and TNF-α) was significantly downregulated by EA 575^®^-treated mMoDCs, regardless of whether they were treated with both concentrations of EA 575^®^ or only the lower concentration (TNF-α; *p* < 0.001). The pattern of Th2 cytokine production aligned with the results observed at the intracellular level. Specifically, EA 575^®^-treated mMoDCs, pretreated with the lower concentration of the extract, increased the production of IL-4 (*p* < 0.05) and IL-5 (*p* < 0.05), while no changes were observed with the higher concentration of EA 575^®^. Conversely, the production of IL-13 was significantly reduced with higher concentrations of EA 575^®^ (*p* < 0.05), whereas lower concentrations had no significant effects. The production of Th17 cytokines (IL-17A and IL-22), Th21 cytokine (IL-21), and Th9 cytokine (IL-9) was significantly decreased in co-cultures with EA 575^®^-treated mMoDCs, regardless of the concentration of the extract used. For IL-17A, IL-21, and IL-9, the effects were dose-dependent. Furthermore, EA 575^®^-treated mMoDCs pretreated with the lower concentration of the extract reduced the production of IL-6 (*p* < 0.05) but increased IL-10 (*p* < 0.01).

### 3.7. Effect of EA 575^®^-Treated mMoDCs on the Development of Treg Populations

**Figure 9:**
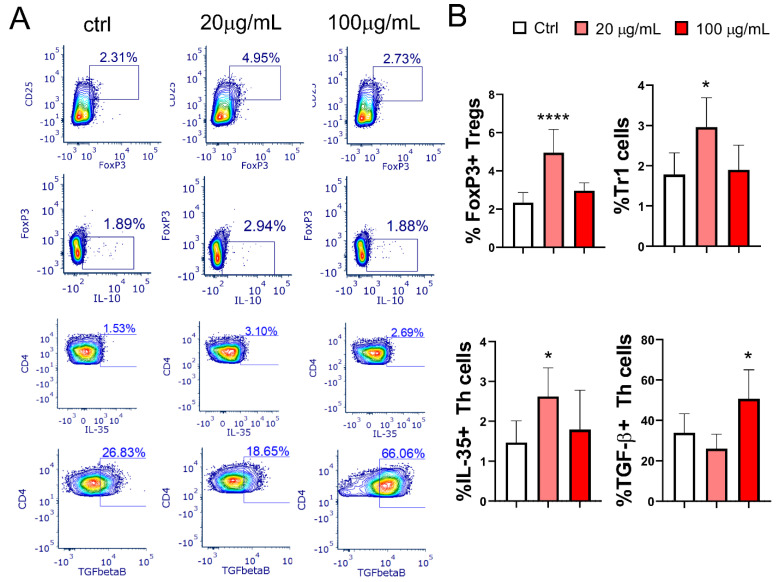
The effect of EA 575^®^-treated mMoDCs on Tregs. Control and EA 575^®^-treated mMoDCs were co-cultured with purified T cells as described in the Section 2. Treg subsets were determined by flow cytometry based on the expression of characteristic intracellular markers. (**A**) Representative plots of one experiment. (**B**) Mean percentages or positive cells ± SD (*n* = 6 donors). * *p* < 0.05; **** *p* < 0.0001 compared to the control (Ctrl).

mMoDCs treated with lower concentrations of EA 575^®^ significantly increased the percentages of Treg subsets, including CD4+CD25hiFoxp3+ Tregs (*p* < 0.0001) and Tr1 cells (Foxp3-IL-10+) (*p* < 0.05), in co-culture with T cells. Additionally, the percentage of CD4+IL-35+ Tregs was also elevated (*p* < 0.05) with lower concentrations of EA 575^®^. In contrast, an increase in CD4+TGF-β+ cells was observed only when MoDCs were treated with a higher concentration of EA 575^®^ (*p* < 0.05) (Figure 9).

### 3.8. Effect of EA 575^®^-Treated mMoDCs on the Development of Exhausted PD1+CD4+ Cells and Cytotoxic CD4+ T Cells

**Figure 10:**
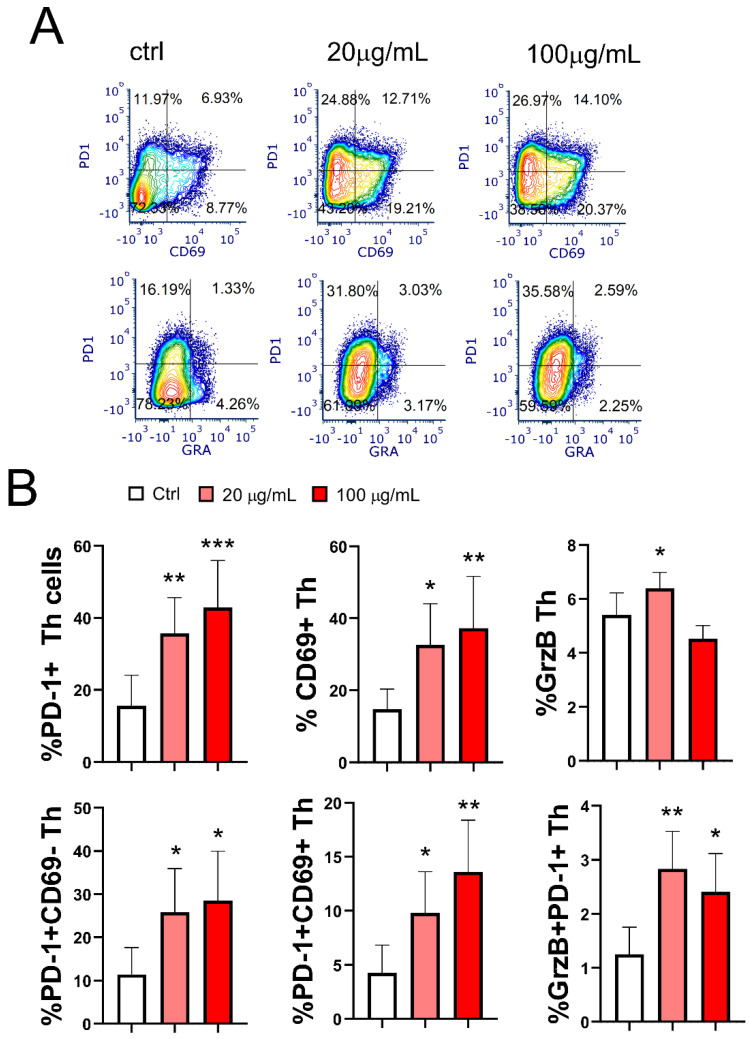
Effect of EA 575^®^-treated mMoDCs on the development of exhausted PD1+CD4+ T cells and cytotoxic CD4+granzyme B+ cells. Control and EA 575^®^-treated mMoDCs were co-cultured with purified T cells as described in the Section 2. The subsets of exhausted Th cells and cytotoxic Th cells were identified by flow cytometry based on the expression of characteristic markers. (**A**) Representative flow cytometry plots from one experiment. (**B**) Mean percentages of positive cells ± SD (*n* = 6 donors). * *p* < 0.05; ** *p* < 0.01; *** *p* < 0.001 compared to the control (Ctrl).

The final aim of this study was to investigate the effect of EA 575^®^-treated mMoDCs on the development of hyporesponsive/exhausted Th cells. As shown in Figure 10, the pretreatment of MoDCs with both concentrations of EA 575^®^ resulted in an increased frequency of PD1+CD4+ T cells (*p* < 0.01 and *p* < 0.001, respectively) with the exhausted phenotype, and this effect was dose-dependent. The proportion of total CD69+ Th cells, as well as a subset of these cells co-expressing PD1 (CD4+PD1+CD69+ cells), followed the same expression pattern in co-culture with EA 575^®^-treated mMoDCs (*p* < 0.05 and *p* < 0.01, respectively).

A distinct population of cytotoxic CD4+ T cells expressing granzyme B was observed in a relatively small proportion of CD4+ T cells in the control mMoDC/T-cell co-culture (5.4 ± 0.8). Interestingly, mMoDCs pretreated with a lower concentration of EA 575^®^ increased the frequency of granzyme B+CD4+ T cells (*p* < 0.05). However, the frequency of a subset of these cells expressing PD1 was significantly increased in the co-culture of T cells with mMoDCs pretreated with both concentrations of EA 575^®^ (*p* < 0.01 and *p* < 0.05, respectively).

## 4. Discussion

Ivy (*Hedera helix* L.) leaf extract contains biologically active compounds with clinical benefits, particularly in managing bronchial infections due to its potential antispasmodic, bronchodilating, and antitussive properties [1,2,5,7,30]. The anti-inflammatory potential of the extract, especially EA 575^®^ [5], prompted an investigation into its effects on adaptive immunity. Using an in vitro co-culture system of MoDCs and T cells, we tested this hypothesis.

Initial experiments assessed the cytotoxicity of EA 575^®^ at various concentrations. Monocytes induced to differentiate into MoDCs remained viable at concentrations up to 100 µg/mL, but higher concentrations (≥200 µg/mL) significantly reduced cell viability due to apoptosis, linked to oxidative stress. Subcytotoxic concentrations (100 µg/mL) caused oxidative stress without inducing apoptosis or autophagy. These results align with reports that higher concentrations of *Hedera helix* extracts induce apoptosis in normal and malignant cells via pro-oxidant activity, with effects varying by experimental conditions and extract composition [31,32,33,34,35]. As this study aimed to identify non-cytotoxic concentrations for further research, detailed mechanisms of cytotoxicity were not explored.

The first experiments examined whether EA 575^®^ affects MoDC differentiation. Key markers of imMoDC differentiation in the presence of IL-4 and GM-CSF include downregulated CD14, upregulated CD1a, HLA-DR, co-stimulatory molecules (CD86, CD40), CD206, CD209, and Dectin 1, alongside tolerogenic markers (PD-1L, ILT3, ILT4, IDO1) [36,37,38]. Our results showed that EA 575^®^ inhibited MoDC differentiation, reducing most differentiation and immunogenic markers (except CD40 and CD14) while upregulating tolerogenic markers (excluding IDO1) in a dose-dependent manner. EA 575^®^-treated MoDCs displayed a tolerogenic phenotype with reduced immune-stimulatory capacity, indicating the significant impact of EA 575^®^ on their maturation potential.

To stimulate maturation, we used LPS and IFN-γ to activate the control and EA 575^®^-treated imMoDCs, generating Th1-polarizing dendritic cells (DCs) with strong immunostimulatory properties [39]. Mature MoDCs (mMoDCs) showed increased CD83, HLA-DR, co-stimulatory molecules (CD86, CD40), and Dectin-1, alongside reduced CD206 and CD209 [28]. They also produced IL-12, IL-23, IL-27, IL-1β, and TNF-α, consistent with robust immune activation [40,41,42].

EA 575^®^ inhibited mMoDC phenotypic and functional maturation in a dose-dependent manner, reducing HLA-DR, CD83, co-stimulatory molecules, and antigen uptake markers (Dectin-1, CD206, CD209). The reduced expression of co-stimulatory molecules was consistent with a diminished allostimulatory potential, as reflected by a decreased proliferation of allogeneic T cells in co-culture with EA 575^®^-treated mMoDCs.

The explanation for the downregulation of other markers is more complex. Dectin-1, crucial for DC maturation, likely represents an additional target of EA 575^®^’s suppressive effect [43]. Reduced CD206, involved in antigen processing and tolerance, and CD209, key for pathogen recognition and DC/T-cell interactions, further contributed to decreased pro-inflammatory and immune responses [44,45,46].

MoDC maturation relies on glycolysis activation via the Phosphatidylinositol 3-kinase/Akt/Mechanistic Target of Rapamycin (PI3K/Akt/mTOR) pathway and p38 Mitogen-Activated Protein Kinase (MAPK), Extracellular Signal-Regulated Kinases 1 and 2 (ERK1/2), and Signal Transducer and Activator of Transcription 3 (STAT3) signaling, antagonized by AMP-Activated Protein Kinase (AMPK), which promotes oxidative phosphorylation via Peroxisome Proliferator-Activated Receptor Gamma (PPARγ) [47,48]. EA 575^®^ components likely interfere with this metabolic switch. Flavonoids, such as rutin, a dominant EA 575^®^ polyphenol, suppress mTOR activity and target Phosphatidylinositol 3-Kinase (PI3K), MAPK, and NF-κB pathways [49,50,51].

Saponins in EA 575^®^, including α-hederin and hederacoside C, modulate signaling pathways like PI3K/Akt/mTOR, MAPK, and AMPK. α-Hederin induces autophagic cell death via ROS-dependent AMPK/mTOR signaling, while hederacoside C inhibits MAPK and NF-κB pathway components [52,53,54,55]. Although the effects of *Hedera helix* extract and its components on DCs remain unexplored, it is reasonable to postulate that most of these signaling pathways are involved. In this context, future studies should address the specific roles of individual components in modulating adaptive immune responses. However, it is also important to emphasize that the extract as a whole is an active and effective medicinal product, rather than any single isolated component within it.

MoDCs treated with EA 575^®^ and matured with LPS/IFN-γ remain semimature, as indicated by their phenotypic markers and reduced ability to stimulate T-cell proliferation. Despite this, these cells modulate Th polarization, inhibiting Th1, Th17, Th9, and Th21 responses in a generally dose-dependent manner. These effects correlate with the decreased production of IL-12, IL-23, and IL-27, key cytokines for Th1 and Th17 responses. These findings are particularly significant, as the Th1, Th17, and Th21 immune responses are linked to chronic inflammation, including autoimmune disorders and obesity-induced asthma. In addition, Th9 cells have a key role in the development of allergic airway inflammation [56,57]. By mitigating these T cell-mediated immune activities, EA 575^®^ emerges as a promising therapeutic candidate.

The explanation of the effect of EA 575^®^-treated MoDCs on the Th2 response, primarily involved in defense against helminthic infections, humoral immunity, and the pathogenesis of allergic diseases such as asthma [58], is more complex. MoDCs treated with lower extract concentrations enhanced the Th2 response, evidenced by increased IL-4 and IL-5 production, whereas higher concentrations suppressed IL-13. This shift may result from the suppression of Th1 and Th17 responses or the activation of genes favoring Th2 differentiation [59]. However, the implications of these findings for atopic asthma remain unclear. In this context, further studies using alternative in vitro immunological models are necessary before proceeding to clinical trials.

Lower concentrations of EA 575^®^ also reduced IL-6 and TNF-α in co-cultures, consistent with previous studies on EA 575^®^’s anti-inflammatory effects [9,10,11,12,13,55]. Hederacoside C, the dominant saponin in EA 575^®^ [11,29], downregulates IL-6, IL-1β, and TNF-α expression while upregulating IL-10. Rutin inhibits TNF-α, IL-6, IL-1β, and cyclooxygenase-2 via NF-κB/MAPK suppression, while chlorogenic acid and dicaffeoylquinic acids reduce inflammation and oxidative stress through similar mechanisms [60,61,62]. Given that various signaling pathways are triggered by EA 575^®^ components [47,48,49,50,51], except for NF-κB as presented for IL-6 [13], their potential role in cytokine modulation warrants investigation in future studies.

Another very important phenomenon that arose from this study is related to the development of tolerogenic DCs in the presence of EA 575^®^. Tolerogenic DCs expressed numerous inhibitory markers including PD-L1, ILT3, ILT4, and IDO1 (evaluated in this study), as well as Inducible Costimulator Ligand (ICOS-L) and Cytotoxic T Lymphocyte Antigen 4 (CTLA-4). Additionally, tolerogenic DCs produce immunoregulatory cytokines IL-10 and TGF-β [63]. Our results showed that EA 575^®^ upregulated PD-L1, ILT3, and ILT4 expression on both imMoDCs and mMoDCs, mainly in a dose-dependent manner. A lower extract concentration also upregulated IDO1 expression in mMoDCs.

The expression of PD-1L and its receptor PD-1 is a physiological process that controls immune responses and inflammation, thereby protecting normal tissue from damage [64]. Increased ILT3 and ILT4 expression on DCs signifies heightened tolerogenic properties [65], with ILT3 being particularly critical for inducing CD4+ Foxp3+ Tregs [66]. The coordinated expression of tolerogenic markers is closely interconnected. For example, tryptophan catabolism via IDO activity induces T-cell nonresponsiveness and tolerance while simultaneously upregulating ILT3 and ILT4 on DCs [67]. Furthermore, the increased expression of IDO1 in DCs is mediated by CTLA-4 [68].

A key function of tolerogenic DCs is the induction of Tregs. Our study showed that mMoDCs treated with lower EA 575^®^ concentrations during differentiation induced classical Treg subsets (CD4+CD25hiFoxP3+), Tr1 cells (CD4+IL-10+FoxP3-), and CD4+IL-35+ Tregs in mMoDC/T-cell co-cultures. These findings correlated with increased IL-10 levels in co-culture supernatants, representing novel observations. Treg subsets differ but act collectively to maintain immune homeostasis. FoxP3+ Tregs inhibit effector Th1, Th2, and Th17 responses depending on involved ligands and transcription factors, contributing to self-tolerance [69]. Tr1 cells, through high IL-10 production, dominantly mediate peripheral tolerance [70], while IL-35+ Tregs secrete IL-35, an immunosuppressive cytokine crucial for their induction and regulatory effects [70,71,72].

Some other findings may relate to the development of tolerogenic DCs by EA 575^®^. For instance, increased CD11c expression by EA 575^®^-MoDCs, observed in our study, has been reported in tolerogenic intestinal DCs in coeliac disease patients [73]. However, this contrasts with the reduced HIF-1α expression by EA 575^®^-MoDCs. HIF-1α regulates immune checkpoint molecules such as PD-L1 and CD73 [74] and is relevant for DCs, as IL-10 production correlates with HIF-1α expression [75].

Initially identified as an early T-cell activation marker, CD69 exhibits both immunosuppressive and pro-inflammatory effects, depending on its interaction with specific ligands such as Gal-1, calprotectin (S100A8/S100A9), Myl9/12, or ox-LDL [76]. CD69 binding to Gal-1 on DCs induces IL-10+ T cells with anti-inflammatory properties [76], while its interaction with Myl9/12 recruits activated T cells to inflamed lungs in asthma [77].

PD-1, another T-cell activation molecule, balances immunity and tolerance. Expressed by Tregs and other immune cells, PD-1 inhibits effector T-cell activation during prolonged antigen stimulation, preventing self-reactive responses [78]. Sustained PD-1 expression drives T-cell exhaustion, characterized by reduced cytokine production and increased inhibitory receptor expression [79,80,81,82]. In our model, EA 575^®^-treated MoDCs increased CD69 and PD-1 expression on CD4+ T cells, elevating the frequency of PD1+CD69+ and PD1+CD69- CD4+ T cells. However, this reflects only the phenotypic characteristics of these cells, and their exhausted function requires further investigation. Additionally, these MoDCs increased the frequency of CD4+Granzyme B+ cells, a subset of cytotoxic CD4+ T cells that may enhance anti-tumor immunity [83]. However, the elevated expression of PD1 on these cells suggests potential exhaustion. Although the increased PD1 expression on these T cell subsets implies hypofunctional properties, the exact role of exhausted T cells in limiting immune responses remains to be elucidated.

Cumulatively, our results significantly expand the understanding of the biological and immunological effects of *Hedera helix* extract, especially EA 575^®^, one of the most standardized and clinically validated extracts. While the study has limitations, such as not investigating CD8+ T cells and not exploring further mechanisms due to the extract’s mixed components, it opens exciting avenues for future research. This includes designing immunological protocols for animal models and clinical studies, particularly for chronic lung diseases, where EA 575^®^ is prominently used, including autoimmune-related conditions.

## 5. Conclusions

This study demonstrates for the first time that EA 575^®^, a dry extract of ivy leaf, is a potent immunomodulator. The extract modulates T-cell immune responses through complex mechanisms involving DCs. EA 575^®^ inhibits the differentiation and maturation of MoDCs, leading to the acquisition of tolerogenic properties. MoDCs treated with EA 575^®^ decrease the production of immunogenic cytokines (e.g., IL-12 family) and reduce T-cell proliferation. Additionally, they inhibit Th1, Th17, Th9, Th21, and pro-inflammatory immune responses. The Th2 response is modulated depending on the specific Th2 cytokine and EA 575^®^ concentration. These immunomodulatory effects are linked to the induction of various subsets of tolerogenic and exhausted Th cells. Lower concentrations of EA 575^®^ are more immunomodulatory, while higher concentrations are more anti-inflammatory. These findings shed light on the immunological activity of EA 575^®^, providing insights into the beneficial effects of *Hedera helix* leaf extract in chronic lung diseases. Moreover, they highlight that EA 575^®^’s activation of immunoregulatory mechanisms may help prevent excessive immune activation, limit inflammation and tissue damage, and maintain immune homeostasis.

## Data Availability

All data are included in this article.

## Supplementary Materials

The following supporting information can be downloaded at: https://www.mdpi.com/article/10.3390/pharmaceutics17060773/s1, Figure S1: Upper row: (A) Expression of ROS in control monocytes induced to differentiate into imMoDCs (left) and EA 575^®^-treated monocytes exposed to 25 µg/mL (middle) or 100 µg/mL (right) of the extract. Histograms are shown for one representative experiment. (B) Values are expressed as the percentage of DHE+ cells (mean ± SD; n = 3). * *p* < 0.05 compared to control (Ctrl). Bottom row: (A) Autophagy in control monocytes induced to differentiate into imMoDCs (left), EA 575^®^--treated monocytes exposed to 25 µg/mL (middle), or 100 µg/mL (right) of the extract. Histograms of LC3II+ cells are shown for one representative experiment. (B) Induction ratios, calculated as the expression levels in the presence versus absence of bafilomycin, are presented as mean ± SD (*n* = 3); Figure S2: The effect of EA 575^®^ on the expression of differentiation and maturation markers on mMoDCs; Figure S3: The effect of EA 575^®^ on the expression of intracellular cytokines in mMoDCs; Table S1: The effect of EA 575^®^ on the production of cytokines by imMoDCs; Table S2: Modulatory Effects of EA 575^®^ on Cytokine Production by imMoDCs and mMoDCs.
