# Supplementary material for: Ivy Leaf Dry Extract EA 575® Is a Potent Immunomodulator Acting on Dendritic Cells
Source: Pharmaceutics. 2025 Jun 12;17(6):773. doi: 10.3390/pharmaceutics17060773 (PMC12196112; doi:10.3390/pharmaceutics17060773)
Supplement: Supplementary file 1 [file pharmaceutics-17-00773-s001.zip › pharmaceutics-3652916-supplementary.pdf]

Supplementary materials

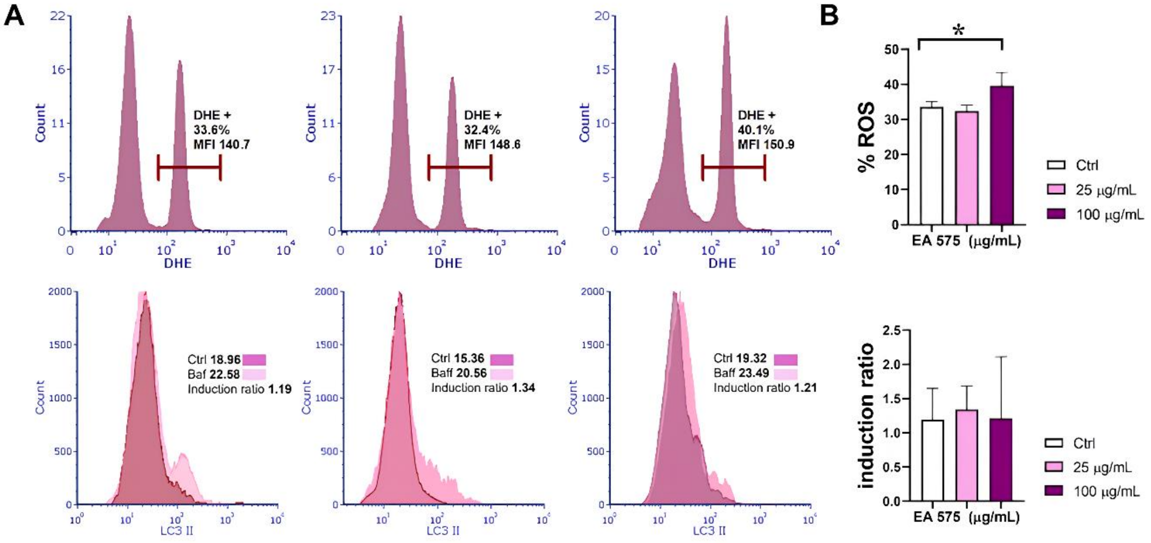

Supplementary Figure S1. Upper row:
A) Expression of ROS in control monocytes induced to differentiate into imMoDCs (left) and EA 575®-treated monocytes exposed to 25 µg/mL (middle) or 100 µg/mL (right) of the extract. Histograms are shown for one representative experiment.
B) Values are expressed as the percentage of DHE+ cells (mean ± SD; n = 3). *p < 0.05 compared to control (Ctrl).

Bottom row:
A) Autophagy in control monocytes induced to differentiate into imMoDCs (left), EA 575®--treated monocytes exposed to 25 µg/mL (middle), or 100 µg/mL (right) of the extract. Histograms of LC3II+ cells are shown for one representative experiment.
B) Induction ratios, calculated as the expression levels in the presence versus absence of bafilomycin, are presented as mean ± SD (n = 3).

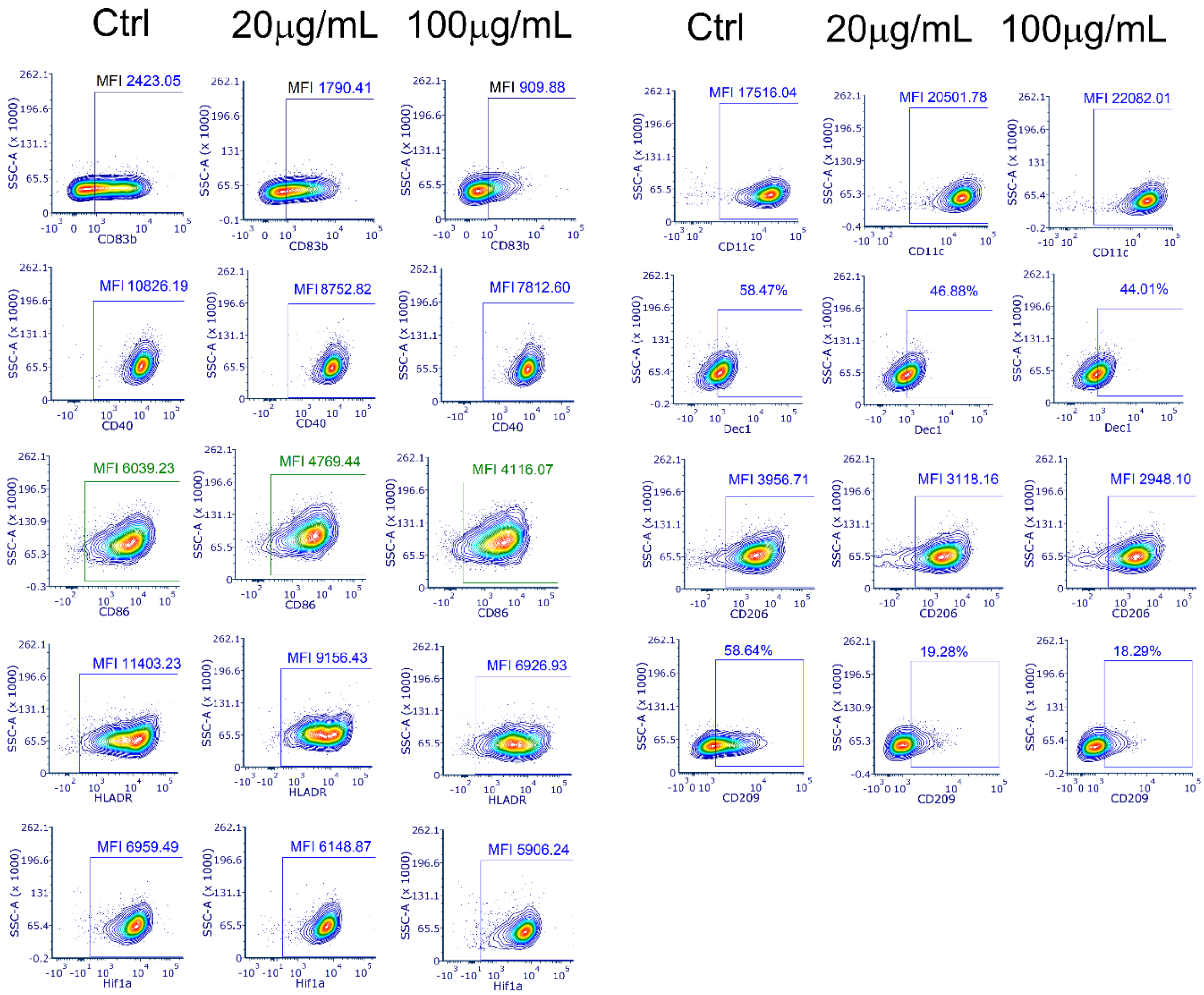

Supplementary Figure S2. The effect of EA 575® on the expression of differentiation and maturation markers on mMoDCs

MoDCs were differentiated from monocytes in the presence of lower (20 µg/mL) and higher (100 µg/mL) concentrations of EA 575® over 4 days and subsequently induced to mature using LPS/IFN-γ. The expression of markers on mMoDCs was analyzed by flow cytometry and is displayed as individual plots from one representative experiment. Results are expressed as the percentage of marker-positive cells or as the mean fluorescence intensity (MFI).

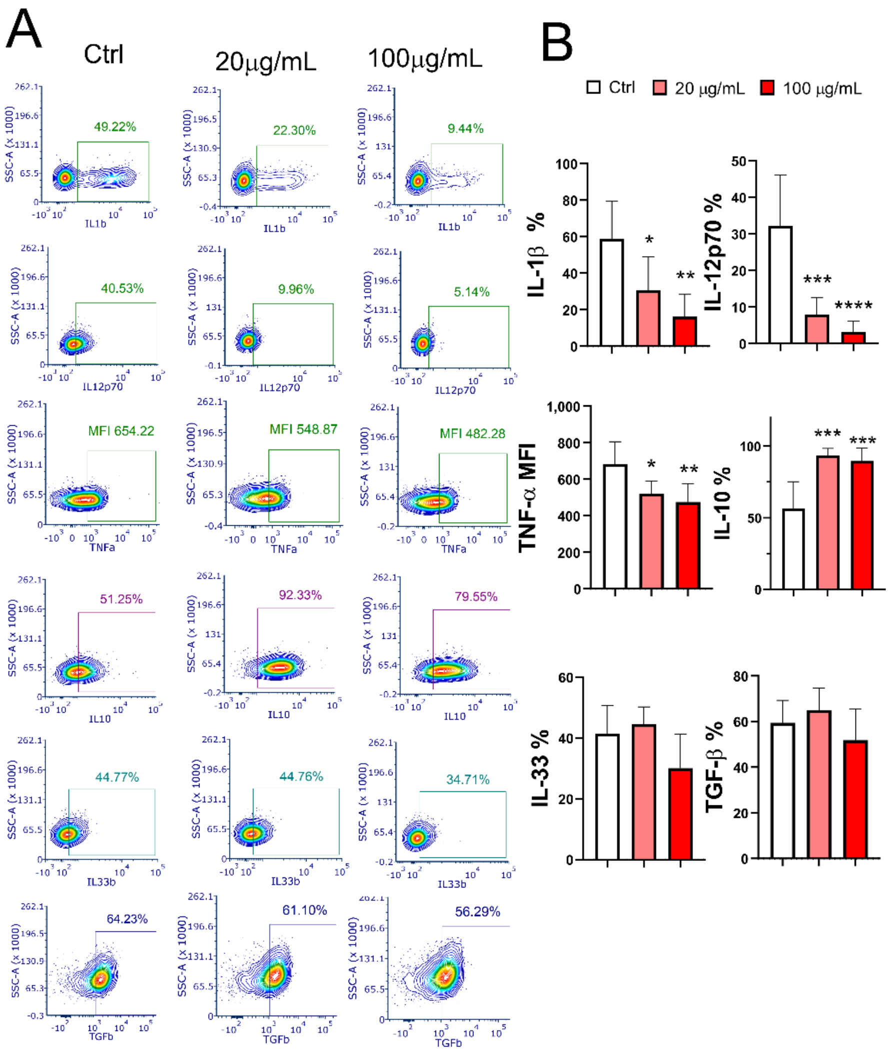

Supplementary Figure S3. The effect of EA 575® on the expression of intracellular cytokines in mMoDCs

MoDCs were differentiated from monocytes in the presence of lower (20 μg/mL) and higher (100 μg/mL) concentrations of EA 575® over 4 days and subsequently induced to mature with LPS/IFN-γ. Cytokine expression in mMoDCs is presented as individual flow cytometry plots from one representative experiment (A) or as mean percentages or mean fluorescence intensity (MFI) ± SD (n = 6 donors) (B). Statistical significance compared to control mMoDCs is indicated above the bars; NS = not significant. *p < 0.05; **p < 0.01; ***p < 0.001; ****p < 0.0001 compared to control (Ctrl).

Supplementary Table S1. The effect of EA 575® on the production of cytokines by imMoDCs

Cytokine (pg/mL)	Control	EA 575® (20 µg/mL)	p	EA 575® (100 µg/mL)	p
IL-12p70	12.1 ± 6.5	10.2 ± 7.1	NS	6.2± 5.2	NS
Il-23	21.3 ± 8.5	22.2 ± 13.0	NS	19.4 ± 10.6	NS
IL-27	96.5 ± 18.3	90.4 ± 21.6	NS	144.8 ± 31.4	< 0.001
IL-10	18.3 ± 14.4	31.6 ± 20.6	NS	29.2 ± 18.8	NS
TGF-β	54.2 ± 21.1	65.2 ± 14.9	NS	74.0 ± 22.9	NS
IL-1 β	13.5 ± 6.3	18.2 ± 4.8	NS	8.9 ± 5.2	NS
TNF-α	19.0 ± 3.9	13.8 ± 3.7	NS	5.7 ± 4.2	< 0.0001
IL-6	188.4 ± 29.8	166.5 ± 28.6	NS	124.5 ± 35.7	< 0.01
IL-8	245.2 ± 45.2	324.6 ± 23.4	NS	126.3 ± 42.3	< 0.0001

MoDCs were differentiated from monocytes in the presence of lower (20 µg/mL) and higher (100 µg/mL) concentrations of EA 575® over a 5-day period.
Cytokine levels were measured in culture supernatants and are expressed as mean concentrations (pg/mL) ± SD (n = 6 donors). NS = not significant.

Supplementary Table S2. Modulatory Effects of EA 575® on Cytokine Production by imMoDCs and mMoDCs

Cytokines	EA 575® (20 µg/mL)		EA 575® (100 µg/mL)	
	imMoDCs	mMoDCs	imMoDCs	mMoDCs
IL-12p70	Ø	↓	Ø	↓
IL-23	Ø	↓	Ø	↓
IL-27	Ø	Ø	↑	↓
IL-10	Ø	Ø	Ø	Ø
IL-8	Ø	Ø	↓	↑
TGF-β	Ø	Ø	Ø	Ø
IL-1β	Ø	↓	Ø	↓
TNF-α	Ø	↓	↓	↓
IL-6	Ø	Ø	↓	↓

Symbols: Ø = no effect; ↓ = decrease ; ↑ = increase
